# An Artificial Neural Network Embedded Position and Orientation Determination Algorithm for Low Cost MEMS INS/GPS Integrated Sensors

**DOI:** 10.3390/s90402586

**Published:** 2009-04-15

**Authors:** Kai-Wei Chiang, Hsiu-Wen Chang, Chia-Yuan Li, Yun-Wen Huang

**Affiliations:** Department of Geomatics, National Cheng-Kung University / No.1, Ta-Hsueh Road, Tainan 701, Taiwan; E-Mails: kwchiang@mail.ncku.edu.tw; cynaomi@gmail.com; jacques207@gmail.com

**Keywords:** GPS, INS, Integration, Mobile Mapping Systems, Artificial Neural networks

## Abstract

Digital mobile mapping, which integrates digital imaging with direct geo-referencing, has developed rapidly over the past fifteen years. Direct geo-referencing is the determination of the time-variable position and orientation parameters for a mobile digital imager. The most common technologies used for this purpose today are satellite positioning using Global Positioning System (GPS) and Inertial Navigation System (INS) using an Inertial Measurement Unit (IMU). They are usually integrated in such a way that the GPS receiver is the main position sensor, while the IMU is the main orientation sensor. The Kalman Filter (KF) is considered as the optimal estimation tool for real-time INS/GPS integrated kinematic position and orientation determination. An intelligent hybrid scheme consisting of an Artificial Neural Network (ANN) and KF has been proposed to overcome the limitations of KF and to improve the performance of the INS/GPS integrated system in previous studies. However, the accuracy requirements of general mobile mapping applications can’t be achieved easily, even by the use of the ANN-KF scheme. Therefore, this study proposes an intelligent position and orientation determination scheme that embeds ANN with conventional Rauch-Tung-Striebel (RTS) smoother to improve the overall accuracy of a MEMS INS/GPS integrated system in post-mission mode. By combining the Micro Electro Mechanical Systems (MEMS) INS/GPS integrated system and the intelligent ANN-RTS smoother scheme proposed in this study, a cheaper but still reasonably accurate position and orientation determination scheme can be anticipated.

## 1. Introduction

The early development of mobile mapping system (MMS) was restricted to applications that permitted the determination of the elements of exterior orientation from existing ground control [1]. According to [2], mobile mapping refers to a means of collecting geospatial data using mapping sensors that are mounted on a mobile platform. Research concerning mobile mapping dates back to the late 1980s. This process is mainly driven by the need for highway infrastructure mapping and transportation corridor inventories. In the early nineties, advances in satellite and inertial technology made it possible to think about mobile mapping in a different way. Instead of using ground control points as references for orienting the images in space, the trajectory and attitude of the imager platform could now be determined directly [3]. Cameras, along with navigation and positioning sensors, are integrated and mounted on a land vehicle for mapping purposes. Objects of interest can be directly measured and mapped from images that have been georeferenced using navigation and positioning sensors.

Direct georeferencing is the determination of time-variable position and orientation parameters for a mobile digital imager [1]. The most common technologies used for this purpose today are satellite positioning using GPS and inertial navigation using an IMU. Although either technology used alone could in principle determine both position and orientation, they are usually integrated in such a way that the IMU is the main orientation sensor, while the GPS receiver is the main position sensor. The orientation accuracy of an IMU is largely determined by the gyro drift rates, typically described by a bias (constant drift rate), the short term bias stability, and the angle random walk [3,4].

In principle, an IMU refers to a set of inertial sensors including three gyroscopes and accelerometers and provides compensated raw measurements including velocities changes (delta-Vs) and orientation changes (delta-θs) along three directions of its body frame. Those who require real time navigation solutions using an IMU require an external computer that has inertial navigation mechanization algorithms. On the other hand, an INS usually refers to an IMU combined with an onboard computer so it can directly provide navigation solutions in real time in the chosen navigation frame. In addition, it also provides compensated raw measurements. Therefore, the main distinction between an IMU and INS is that the former alone can’t provide real time navigation solutions. It only provides compensated inertial measurements, while an INS alone can provide real time navigation solutions, as well as compensated inertial measurements [5].

**Figure 1.**
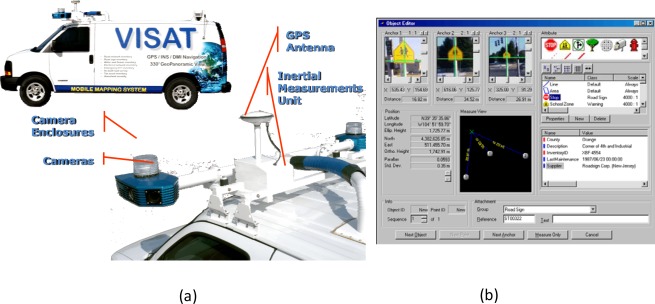
(a) An example of land based MMS (b) An example of direct geo-referencing an object of interest (Adopted from [1]).

The development of land based MMS was initiated by two research groups in North America, The Center for Mapping at The Ohio State University, USA and the Department of Geomatics Engineering at The University of Calgary, Canada [1,5]. In the early 2000s, a number of land based systems have been applied in commercial applications [5–12]. There had been high expectations that these systems would have a large impact on conventional transportation surveying and mapping [1,2]. Figure 1a illustrates an example of a mobile mapping van and Figure 1b depicts an example of direct geo-referencing the traffic sign of interest from three geo-referenced images provided by a land based MMS. As shown in Figure 1a, the common feature of MMS is that more than one camera is mounted on a mobile platform, allowing for stereo imaging and 3-D measurements. Direct georeferencing of digital image sequences is accomplished through the use of navigation and positioning techniques. Various positioning sensors, GPS, IMU and dead-reckoning, can be combined for data processing to improve the accuracy and robustness of georeferencing. The ground control required for traditional mapping is thus eliminated. As shown in Figure 1b, the coordinates of the traffic sign can be determined directly through three geo-referenced images without any ground control and its attribute can be added to the spatial information database for future use.

The KF approach has been widely recognized as the standard optimal estimation tool for current INS/GPS integration schemes. However, it has limitations, which have been reported by several researchers [13–18]. The major inadequacy related to the utilization of KF for INS/GPS integration is the necessity to have a predefined accurate stochastic model for each of the sensor errors [18]. Furthermore, prior information about the covariance values of both inertial and GPS data as well as the statistical properties (i.e. the variance and the correlation time) of each sensor system has to be accurately known [17]. Furthermore, for INS/GPS integration applications (where the process and measurement models are nonlinear), Extended Kalman filter (EKF) operates under the assumption that the state variables behave as Gaussian Random Variables. Naturally, EKF may also work for nonlinear dynamic systems with non-Gaussian distributions, except for heavily skewed nonlinear dynamic systems, where EKF may experience problems [3].

On the other hand, ANN techniques have been applied to develop alternative INS/GPS integration schemes to overcome the limitations of KF and to improve the positional accuracy of vehicular navigation systems during GPS signal blockages [18]. However, Chiang [18] indicated that future development concerning the use of artificial intelligent techniques such as ANN for INS/GPS integration should include an integrated approach using KF and Artificial Intelligence (AI) (e.g., ANN). Such an integrated approach would have the capability of estimating all navigation states, using the advantages of AI techniques for practical solutions. Goodall *et al*. [19] proposed an ANN-KF hybrid scheme that is capable of estimating all navigation states and which uses the advantages of ANN techniques to successfully improve the positioning accuracy of vehicular navigation systems during GPS signal blockages. None of these previous studies aimed at developing a complete Positioning and Orientation System (POS) to meet the requirements of mobile mapping applications in terms of the available states and achievable accuracy. In fact, the scope of the earlier studies is limited to incorporating ANN to bridge the gap between GPS outages by improving the positioning accuracy for navigation purposes. Therefore, the issues concerning orientation angles have not been discussed thoroughly.

## 2. Problem statements

Post-mission processing, when compared to real-time filtering, has the advantage of having the data of the whole mission to estimate the trajectory. This is not possible when using filtering because only part of the data is available at each trajectory point, except the last one [20]. When filtering is used in the first step, an optimal smoothing method, such as RTS backward smoother, can be applied. It uses the filtered results and their covariances as a first approximation. This approximation is improved by using additional data that was not used in the filtering process. Depending on the type of data used, the improvement obtained by optimal smoothing can be considerable. For most of the surveying applications that require superior accuracy, only data acquisition has to be implemented in real time. On the contrary, data computation and analysis are allowed to be post-processed. General speaking, most of the direct geo-referencing applications require higher accuracy platforms, especially in attitude determination. In addition, the workflows of general mobile mapping applications include data acquisition, georeferencing, measurement and Geographical Information System (GIS) processing [1]. Only data acquisition can be implemented in real time for the collection of IMU, GPS and CCD image data. For georeferencing process which puts POS stamps on images and measurement process that obtains 3-D coordinates of all important features and stores them in GIS database, only post-mission processing can be implemented based on the complexity of those processes. Therefore, most of the commercially available land based systems operate in real time only for data acquisition and conduct most of the data processing and analysis in post-mission mode [1,2].

Consequently, high accuracy requirements for position and attitude determination in mobile mapping applications can be achieved in post-mission mode with an optimal smoothing algorithm. In fact, most of the commercial systems use an optimal smoothing algorithm to provide accurate position and orientation for direct geo-referencing [3]. As mentioned previously, an ANN-KF hybrid scheme has been proposed to improve the positional accuracy of vehicular navigation systems during GPS signal blockages [19]. In fact, all of the current commercially available INS/GPS integrated POSs use tactical grade or better IMUs to provide accurate POS solutions for general mobile mapping applications. Therefore, upgrading the hardware (e.g., IMU) can be considered an effective solution for improving the accuracy of POS solutions when a low cost MEMS IMU is used. However, such improvement is rather limited as the availability of high grade (navigation) IMUs is regulated by the governmental regulations of certain countries where the IMUs are produced.

The price of available medium grade (tactical grade) IMUs is very high at the present. In certain countries, the availability of medium grade IMUs is limited. Therefore, another effective way to improve the accuracy of a low cost MEMS INS/GPS integrated POS solution is through the improvement of the POS algorithm. Compared to the hardware solution mentioned above, the software solution is cost effective for developing a low cost INS/GPS integrated POS for general mobile mapping applications.

In the present study, a hybrid scheme that adopts both ANN and an optimal smoothing algorithm is proposed for achieving higher accuracy POS parameters for direct geo-referencing applications using a low cost MEMS IMU. The objectives of this study are to : (1) develop an ANN embedded RTS smoother scheme for an INS/GPS integrated POS used for land based mobile mapping applications; (2) verify the performance of the proposed system using a MEMS INS/GPS integrated system in land vehicular environments and (3) compare the performance with a previously developed ANN-KF hybrid scheme.

## 3. From Kalman filtering to optimal smoothing

To estimate navigation solutions optimally, the output of the INS mechanization needs to be integrated with the position and velocity solutions derived from GPS. EKF is the most popular estimation technique for such integration. A simple form of the mechanization equations in the local level frame can be written as follows [2]:

(1) $$\left[\begin{array}{l} {\dot{r}}^{l}\\ {\dot{v}}^{l}\\ {\dot{R}}_{b}^{l} \end{array}\right]=\left[\begin{array}{l} D^{-1}v^{l}\\ R_{b}^{l}\;f^{b}-(2\Omega_{\mathrm{le}}^{l}+\Omega_{\mathrm{el}}^{l})v^{l}+g^{l}\\ R_{b}^{l}(\Omega_{\mathrm{ib}}^{b}+\Omega_{\mathrm{il}}^{b}) \end{array}\right]$$

where *r^l^* is the position vector [*ϕ* (latitude), *λ*(longitude), *h*(height)], *v^l^* is the velocity vector (e, n, u), $R_{b}^{l}$ is the transformation matrix from the IMU body to local frame as a function of attitude components, *g^l^* is the gravity vector in the local level frame, $\Omega_{\mathrm{ib}}^{b}$, $\Omega_{\mathrm{il}}^{b}$ are the skew-symmetric matrices of the angular velocity vectors $w_{\mathrm{ib}}^{b}$, $w_{\mathrm{il}}^{b}$ respectively, *D*^−1^ is a 3×3 matrix whose non-zero elements are functions of the user’s latitude *ϕ* and ellipsoidal height (h).

For further discussion concerning the solutions and numerical implementations of the above differential equation, see El-Sheimy [3]. An INS mechanization algorithm by itself seldom has good performance due to the inertial sensor biases and the fixed-step integration errors that make the POS parameters diverge quickly. The navigation software must have some approach to account for these error sources to correct the estimated POS parameters [21]. The dynamic error model used in KF for the navigation parameters (position, velocity, and attitude) can be determined through the linearization of the INS mechanization equations and by neglecting insignificant terms in the resultant linear model. A simplified form is then obtained as [22]:

(2) $$\begin{array}{l} \delta {\dot{r}}^{l}=D^{-1}{\delta v}^{l}\\ \delta {\dot{v}}^{l}=-({2\Omega }_{\mathrm{ie}}^{l}+\Omega_{\mathrm{el}}^{l})\times {\delta v}^{l}-{\delta R}_{b}^{l}\;f^{b}+R_{b}^{l}\;\delta f^{b}+\delta g^{l}\\ \delta {\dot{A}}^{l}=E{\delta v}^{l}+R_{b}^{l}{\delta w}^{b}\\ {\delta f}^{b}=b_{a}+\mathrm{diag}(f^{b})s_{a}\\ {\delta w}^{b}=b_{g}+\mathrm{diag}(w^{b})s_{g} \end{array}$$

where δ*r^l^* is the position error state vector in the local level frame, *δv^l^* is the velocity error state vector in the local level frame, *δA^l^* is the attitude error state vector in the local level frame, *δg^l^* is the error in the computed gravity vector in the local level frame, *δf^b^* & *δω^b^* are accelerometer bias and gyro drift vectors in the body frame respectively, and *S_a_* and *S_g_* are scale factors of accelerometers and gyros respectively, and *E* is a 3×3 matrix whose non-zero elements are a function of the vehicle’s latitude and the Earth’s radii of curvatures.

In EKF, INS errors are updated by the differences between GPS and INS solutions. The EKF applied in this study has 21 states [23]:

$${\left[\begin{array}{ccccccc} {\delta r}_{1\times 3} & {\delta v}_{1\times 3} & {\delta A}_{1\times 3} & b_{a,1\times 3} & b_{g,1\times 3} & s_{a,1\times 3} & s_{g,1\times 3} \end{array}\right]}^{T}$$

The equations of KF are divided into two groups of equations: prediction and update. The time prediction equations are responsible for the forward time transition of the current epoch (k−1) states to the next epoch (k) states. The prediction equations are:

(3) $${\hat{x}}_{k}\;\left(-\right)=\Phi_{k}{\hat{x}}_{k-1}\;\left(+\right)$$

(4) $$P_{k}\;\left(-\right)=\Phi_{k}P_{k-1}\;\left(+\right)\;\Phi_{k}^{T}+Q_{k-1}$$

where *x̂* is the optimally estimated state vector, Φ is the state transition matrix, *P* is variance-covariance matrix of inertia states, *Q* is the system noise matrix, (−) is the estimated value after prediction, (+) is the estimated value after updating.

The measurement update equations utilize new measurements into the priori state estimation to obtain an optimized posteriori state estimation. The measurement update equations are given as:

(5) $$K_{k}=P_{k}\;\left(-\right)\;H_{k}^{T}\;{\left[H_{k}P_{k}\;\left(-\right)\;H_{k}^{T}+R_{k}\right]}^{-1}$$

(6) $${\hat{x}}_{k}\;\left(+\right)={\hat{x}}_{k}\;\left(-\right)+K_{k}\;\left(Z_{k}-H_{k}{\hat{x}}_{k}\;\left(-\right)\right)$$

(7) $$P_{k}\;\left(+\right)=P_{k}\;\left(-\right)-K_{k}H_{k}^{T}P_{k}\;\left(-\right)$$

where *K* is the Kalman gain matrix, *H* is the design matrix, *Z* is the vector of updating measurements of position and velocity, *R* is the measurements variance-covariance matrix.

**Figure 2.**
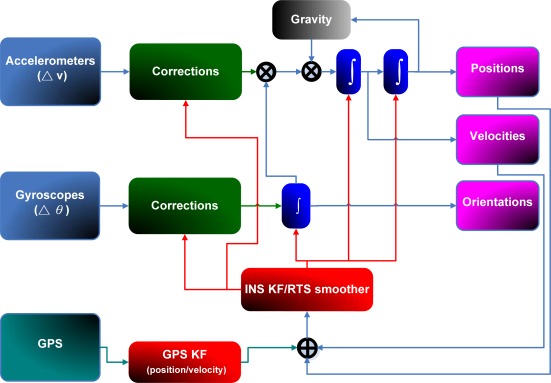
A loosely coupled INS/GPS integration architecture (Closed loop).

The update engine of KF is triggered at every GPS measurement using the difference between GPS and INS solutions as input. Hence, KF generates an updated estimate for reducing the INS errors using measurement update equations. Whenever a GPS measurement is unavailable, KF works in time prediction mode to estimate the error state vector. In this case, the KF equations need the statistical properties of the system to be stationary and well defined, which cannot be guaranteed [23]. Figure 2 shows a closed loop and loosely coupled INS/GPS integration architecture, which is implemented in this study.

As shown in Equation. 6, KF provides the optimal estimate of a state vector at epoch k (*x̂_k_*) by using measurements (updates) that are only available up to epoch k. In contrast, the optimal backward smoothing allows an optimal smoothed estimation of the state vector at epoch k (*x̂_ks_*) utilizing all or some of the measurements that are available after epoch k. The smoothed estimate (*x̂_ks_*) could be considered to be an optimal combination of a forward estimate and a backward estimate. The forward estimate is obtained by using all measurements up to k; it is the estimate provided by KF. The backward estimate is obtained by using all or some of the measurements after k. Since more measurement updates are used for the estimations, the BS estimates in general, if not more accurate, can never be worse than the filtered estimates [20].

**Figure 3.**
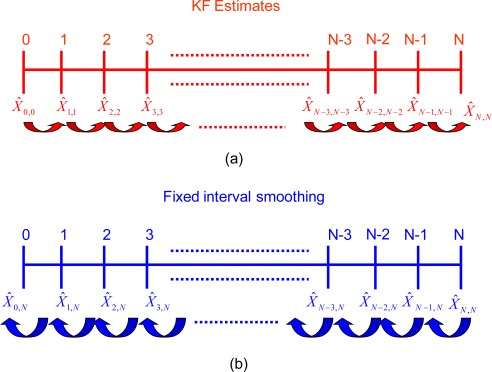
Fixed interval backward smoothing algorithm.

Because smoothing is performed after the filtering stage, all smoothing algorithms depend on the obtained filtered solution. Thus, accurate smoothing requires accurate filtering [20]. According to Gelb [20], there are three types of backward smoothing algorithms; fixed-interval smoothing, fixed-point (single-point) smoothing, and fixed-lag smoothing. Only the fixed interval smoother will be considered in this study; see Gelb [20], and Brown and Hwang [6] for details concerning other smoothing algorithms. As indicated in Figure 3a, DGPS measurements are available at each epoch for the whole mission of time span N. The filtered solution estimates are obtained from the KF at each epoch k (*x̂_k,k_*), where k = 0, 1, 2...N[3]. In fixed-interval smoothing, the initial and final time epochs of the whole interval of measurements (i.e. 0 and N) are fixed. The requirement here is the optimal smoothed estimate at all epochs k in the interval between 0 and N, as indicated in Figure 3b. In this case, all measurement updates between 0 and N are used, so the optimal smoothed estimate at epoch k is ${\hat{x}}_{k,k}^{s}$. Obviously, this type of smoothing can only be carried out in post-mission since it requires the availability of all measurements up to N [23].

RTS backward smoother is implemented in this study. Compared to other fixed-interval smoothers, RTS algorithm is the easiest and simplest to implement [20]. RTS smoother consists of a forward sweep and a backward sweep. The forward sweep is the common KF with all predicted and updated estimates and corresponding covariance saved at each epoch of the whole mission. The backward sweep begins at the end of the forward filter (i.e. at epoch N) with the initial conditions of ${\hat{x}}_{N,N}^{s}={\hat{x}}_{N,N}$ and *P^s^_N,N_* = *P_N,N._* The RTS algorithms are as follows [20]:

(8) $${\hat{x}}_{k,N}^{s}={\hat{x}}_{k,k}+A_{k}\;({\hat{x}}_{k+1,N}^{s}-{\hat{x}}_{k+1,k})$$

(9) $$A_{k}=P_{k,k}\;\Phi_{k+1,k}^{T}\;P_{k+1,k}^{-1}$$

where ${\hat{x}}_{k,N}^{s}$ is the smoothed estimate of the state vector, *A_k_* is the smoothing gain matrix, and k=N−1, N−2…0. The covariance matrix of the smoothed states is given as follows [20]:

(10) $$P_{k,N}^{s}=P_{k,k}+A_{k}\;(P_{k+1,N}^{s}-P_{k+1,k})\;A_{k}^{T}$$

**Figure 4.**
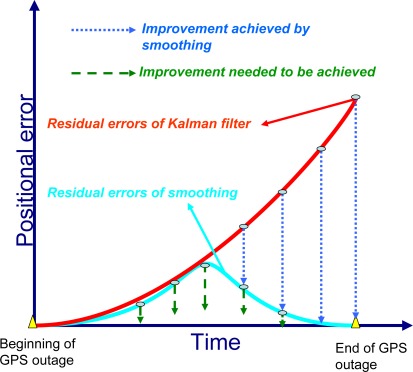
The impact of smoother on the positioning error during GPS outage.

The RTS smoothed estimate at any epoch k is computed as a linear combination of the filtered estimate at that epoch and the smoothed estimate at the heading epoch k+1. Thus, the RTS smoothed estimate can be considered as updating the forward filtered solution to obtain improved estimates [3]. The computation of the smoothed estimate at each epoch requires the storage of the KF predicted and updated (filtered) estimates and their corresponding covariance at each epoch. This is the case in INS/DGPS integrated solutions when uninterrupted data streams are available. In case of DGPS outages, only predicted estimates and covariances are available. A conceptual plot of the impact of the RTS smoother on the positioning error in case of a GPS outage is given in Figure 4. As illustrated in this Figure, the use of a post-mission RTS smoother removes the residual errors of KF significantly; however, some residual errors still remain, so an ANN-RTS smoother scheme is used to reduce the residual errors of the RTS smoother by incorporating the benefits of artificial intelligence.

## 4. The artificial neural networks

In this study, An ANN embedded scheme is implemented to learn and compensate for the residual errors of KF and the RTS smoother, respectively, to improve the accuracy of the attitude angles. The proposed scheme is capable of learning how the state vector (i.e., position or attitude errors) behaves based on the dynamics of the platform and the error characteristics of the inertial sensors being used. The residual error compensation scheme of KF involves a series of complicated non-linear function approximations to adapt to the variations of vehicle dynamics or sensor errors [18]. The ANN is the obvious choice to learn nonlinear functional relationships. In particular, multilayer feed-forward neural networks or radial basis function neural networks are most suited since they are capable of approximating any continuous functional relationship, to any degree of accuracy, if given enough neurons [24].

**Figure 5.**
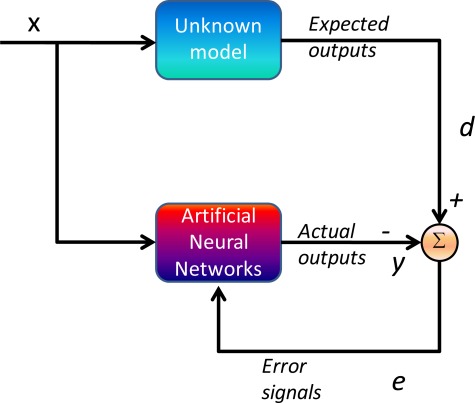
Supervised learning for function approximation.

Although both Multilayer Feed-forward Neural Networks (MFNN) and Radial Basis Function (RBF) neural networks are competent at function approximation, the former are more suitable for sequential on-line applications [24]. RBF neural networks are generally trained in batch mode if trained in sequential mode, the number of basis functions must be fixed ahead of time, which can seriously limit the accuracy of the estimates since radial basis function neural networks often require more neurons/basis functions than MFNN to solve a complicated estimation problem [15]. In contrast, MFNN can fix the architecture of the network ahead of time through empirical experiments. The trained network can then be adjusted whenever new input data is available without changing the architecture of the overall network [24]. In order for MFNN to learn the error behavior of KF, it must first be shown examples of how the errors behave based on the dynamics of the vehicle or platform. This is a form of supervised training whereby the network develops itself based on inputs and observed outputs, as shown in Figure 5.

**Figure 6.**
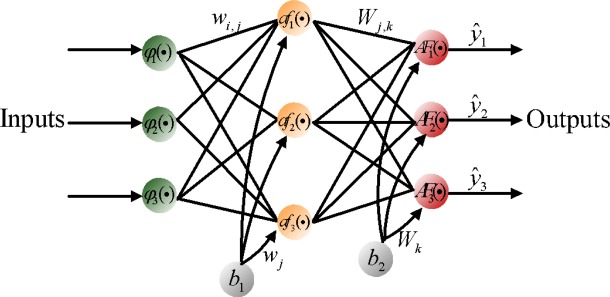
A fully connected Two Layer Feed Forward Network.

The topology of an ANN is constructed from small processing units (neurons) that are interconnected within the network using weighted links. In general, the basic model of the neuron contains three major components: (a) weight links 〈*w_i,j_*,*W_j,k_*〉; (b) an adder for summing the input signals *ϕ_i_* that are weighted by respective synapses of the neuron and external bias (*b_k_*); and (c) an activation function *φ*(•) for limiting the amplitude of the neuron output and the final output *y_k_*. Figure 6 shows a feed forward neural network, which contains external inputs (*ϕ*_1_,*ϕ*_2_,*ϕ*_3_), a hidden layer with 3 hidden neurons, and an output layer with 3 output neurons. The depicted network is said to be fully connected since all inputs/all neurons in one layer are connected to all neurons in the following layer [15]. The mathematical formula for the depicted network can be expressed in the form:

(11) $${\hat{y}}_{i}(t)=\hat{f}(\phi ,\theta )={\mathrm{AF}}_{i}\left[\sum_{j=1}^{n_{h}}W_{j,k}{\mathrm{af}}_{k}\left(\sum_{l=1}^{n_{\phi }}w_{i,j}\phi_{i}+w_{j,0}\right)+W_{k,o}\right]$$

where θ specifies the parameter vector, which contains all the adjustable parameters of the network; i.e., the weights and bias 〈*w_j,l_*,*W_i,j_*〉. Since the bias can be interpreted as a weight acting on an input clamped to 1; i.e., *b*_1_, *b*_2_ = 1,the joint description “weight” will most often be applied covering both weights and bias. To determine the weight values one must have a set of examples of how the outputs, *ŷ_i_*, should relate to the input, *ϕ_l_*. The process of obtaining the weights from these examples is called supervised learning; it is basically a conventional estimation process. That is, the weights are estimated from existing examples in such a way that the network, according to some metric, models the true relationship as accurately as possible. This supervised learning process can be implemented using a backpropagation learning algorithm.

As shown in Figure 6, *af*(•) and *AF*(•) represent the activation functions for hidden neurons and output neurons. In this study, a nonlinear activation function (e.g., hyper tangent) is utilized at the hidden neurons so that the nonlinearities can serve to enhance the network’s approximation capabilities and reduce the impact of noise, while a linear activation function is applied at the output layer neurons. This combination, one nonlinear hidden layer and a linear output layer, has shown the ability to approximate any differentiable function [15].

There are many variations of backpropagation learning algorithms which are used to speed up the training process and avoid local minimums. The variant used in this article is the Levenberg-Marquardt learning algorithm. This method is considered the fastest and most stable among all the available learning algorithms [18].

## 5. An open loop design for ANN-RTS smoother scheme

**Figure 7.**
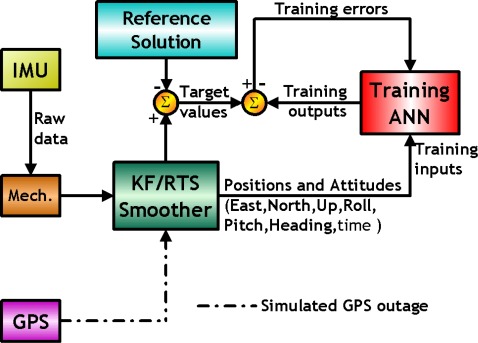
An ANN training architecture.

As shown in Figure 7, KF and RTS smoother are utilized to optimally estimate the position errors, velocity errors, attitude errors, and the sensor biases, and to compensate for their effect in real- time and post-mission modes, respectively. In fact, either approach can provide optimally estimated navigation parameters including position, velocity, and attitudes. In addition, sensor biases and scale factors can be estimated and feedback to the INS mechanization to correct the raw measurements provided by an IMU. However, since the scope of this study is limited to POS parameters, including positions and attitude angles, only the components concerning the POS parameters are shown in Figure 7. To obtain highly accurate POS parameters, an intelligent compensation method can be implemented to predict the error of KF or RTS smoother during GPS signal outages. During the prediction process when no GPS signal is available, the outputs of KF or RTS smoother might contain errors that cannot be estimated well due to the limitations mentioned in Chiang [8]. Consequently, the overall accuracy of the estimated POS parameters can deteriorate. Therefore, an algorithm that can predetermine the error behavior of KF or RTS smoother during GPS outages is needed. Hence, the ANN-KF and ANN-RTS hybrid schemes are proposed.

As indicated in Figure 7, the errors of POS parameters estimated by KF and RTS smoother are used as the desired output or target values during the learning process of the proposed ANN architectures. The POS parameters estimated by KF and RTS smoother along with the time information in each scenario are used as the inputs of the proposed architectures. Since the goal of the proposed schemes is to compensate for the errors of the POS states estimated by KF and RTS smoother during GPS outages, the target values, the POS errors of the KF and RTS smoother, are obtained with intentionally added GPS outages with respect to reference solutions which are generated by the post-mission process (e.g. RTS smoother) using a superior IMU with the full availability of GPS, respectively. In other words, the reference trajectories are generated without intentionally added GPS outages.

**Figure 8.**
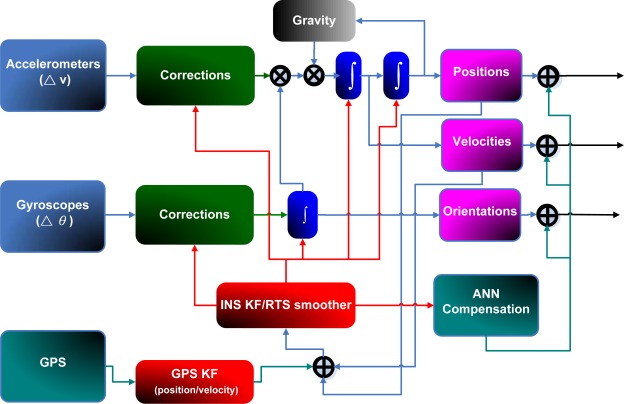
The implementation of ANN embedded KF and RTS smoother.

The parameters of the ANN, including weights and bias are then tuned epoch-by-epoch according to the training error. The training process terminates after the training errors reach the error threshold. Then the ANN estimated attitude errors and desired attitude errors can be considered to be similar. In other words, the ANN based scheme has learnt the error behavior of KF and RTS smoother. This process can be regarded as the training mode of the proposed intelligent scheme. After being well trained, the proposed ANN compensation scheme was added to Figure 2. Consequently, the open loop ANN-KF and ANN-RTS smoother hybrid scheme can be constructed, as shown in Figure 8.

They can be utilized in the compensation or prediction mode when the new measurements are provided by an IMU during GPS outages. Similar to the training mode, the intelligent architectures first receive raw data from an IMU and then use the INS mechanization along 21 states of KF and RTS smoother to estimate POS parameters, respectively. Meanwhile, the estimated POS parameters are sent to the proposed ANN architecture along with time information to generate predicted errors to compensate for the estimated POS parameters provided by KF and RTS smoother simultaneously. Errors of POS parameters are predicted with the proposed ANN scheme. The correction can be completed after the predicted errors have been removed from the outputs of KF and RTS smoother, respectively. It is worth mentioning that the proposed architectures can be operated in real time for compensating attitude errors when KF is used.

**Figure 9.**
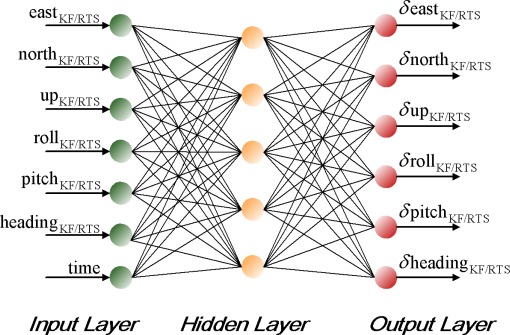
The topology of proposed ANN scheme

The topology of the proposed ANN scheme is shown in Figure 9. The complexity of applying MFNN varies according to the complexity of the application. In contrast, the complexity of ANN depends on its topology, which consists of the number of hidden neurons (size) and hidden layers (depth). An MFNN with an optimal topology is expected to provide the best approximation accuracy to the unknown model using the most appropriate number of hidden neurons and hidden layers. There are many ways to decide on the most appropriate number of hidden neurons; see [24] for details. The common principle indicates that the most appropriate number of hidden neurons is application dependent and can only be decided empirically during the early stages of the topology design. It is very common in the design phase of neural networks to train many different candidate networks that have different numbers of hidden neurons and then to select the best, in terms of its performance based on an independent validation set [15]. In this study, the empirical approach is applied to decide the optimal number of hidden neurons required for the proposed scheme.

## 6. Results and Discussion

**Figure 10.**
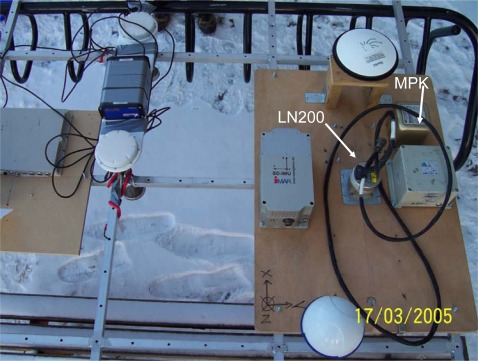
The set up of the tested systems.

**Figure 11.**
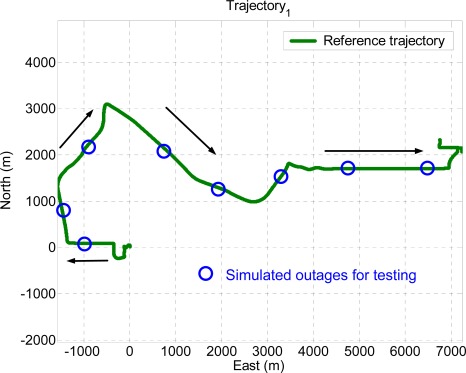
Test trajectory 1.

**Figure 12.**
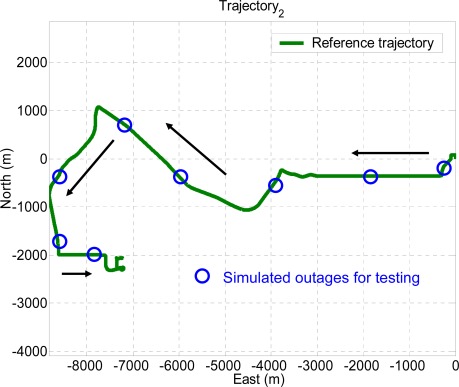
Test trajectory 2.

**Figure 13.**
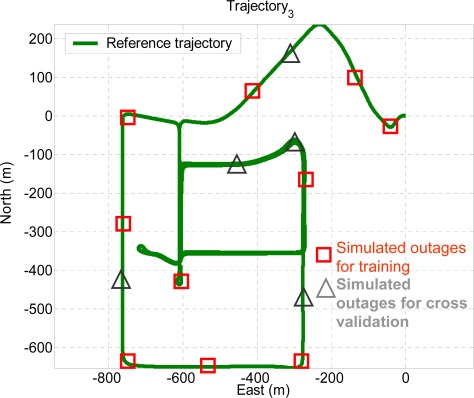
Test trajectory 3.

To evaluate the performance of the proposed schemes, three field tests were used. The tests were conducted in land vehicle environments using different integrated systems consisting of one tactical grade IMU, Litton LN200 (1 deg/hr), a low cost MEMS IMU, BEI MotionPak II, and two NovATel OEM-4 receivers. In this study, those IMUs were applied to collect inertial measurements in the field and then those measurements along with carrier phase DGPS solutions were fed into software that has inertial navigation algorithms and EKF to estimate inertial states optimally. The integrated system with LN200 IMU was used as the reference system. The measurements and navigation solutions provided by the integrated system with MotionPak II were used to verify the performance of proposed schemes. Figure 10 shows the set up of these systems. Figures 11–13 illustrate the trajectories of the field tests. The experimental conditions are summarized in Table 1.

The GPS measurements were processed using GrafNav™ 7.0 software (Waypoint Consulting Inc.) in carrier phase DGPS to achieve ten centimeter level accuracy. The reference trajectories were generated by the integrated system with LN200 IMU. They were generated using 21 states EKF and RTS backward smoothing. The parameters of EKF and the smoother applied in this article were well tuned so that they can represent the best achievable navigation accuracy for tactical grade IMUs.

Ten GPS outages, marked in circles, each 60 seconds in length, were simulated using the measurements collected in the third field test, as indicated in Figure 13. Therefore, the outputs of KF and RTS smoother provided by those systems were applied as the inputs for the proposed architectures, as shown in Figure 8. In addition, the outputs of KF and RTS smoother with simulated outages were then compared with the reference trajectory. The errors, which can be interpreted as the error behavior of KF and RTS smoother, were then applied as the desired output for training. As shown in Figure 13, the dynamic variations experienced by the vehicle during the simulated outages include straight line segments, sharp turns, accelerations and decelerations. It is worth mentioning that five simulated outages, marked by triangles, were used as the independent dataset for cross validation during training process to ensure generalization capability as well as to avoid possible over-training problems.

Sixteen GPS outages in total, each 60 seconds in length, were simulated using the measurements collected in the first and second field test using the integrated system with the MotionPak II (MEMS) IMU. The dynamics variations experienced by the vehicle during the simulated outages are indicated by the blue circles in Figures 11 and 12. Both field test data sets are applied to verify the performance of the proposed schemes.

## 7. The training of the proposed schemes

**Figure 14.**
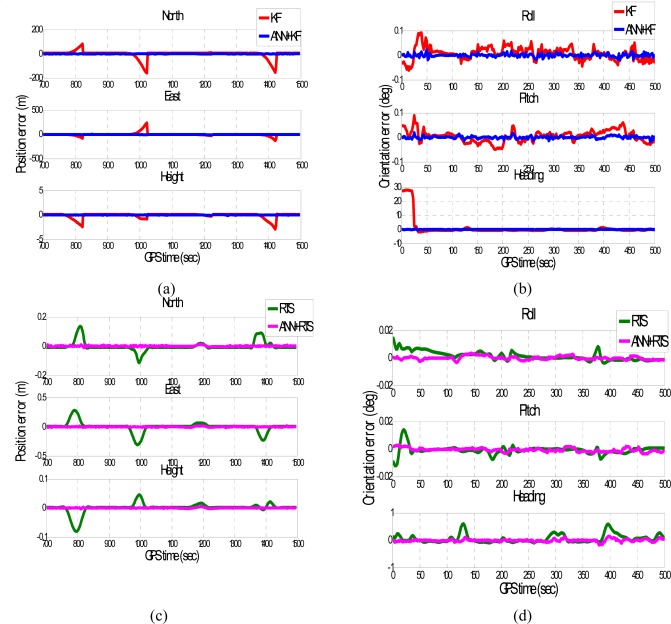
The samples of training results (Trajectory 3).

Figure 14 depicts the samples of the training results in terms of the errors of POS solutions, respectively. Only some of the results are presented in this Figure to illustrate the performance of conventional and proposed schemes in more details. The number of hidden neurons for each scheme was 15, which was decided empirically. Only one hidden layer was used in the proposed schemes. Table 2 summarizes the training results. As shown in Table 2, the column labeled “original” represents the “raw” attitude errors of KF and RTS smoother compared to the reference solutions. Similarly, the column labeled “compensated” represents the “corrected” POS parameters of KF and RTS smoother after the proposed ANN-KF and ANN-RTS smoother schemes were applied compared to the reference solutions, respectively. As indicated in Figure 14 and Table 2, the proposed ANN-KF and ANN-RTS smoother schemes learn the error behavior of KF and RTS smoother well during simulated GPS outages, especially the heading angles and height components.

To show the meaning of the significant improvements presented in this section, the proposed scheme’s ability to catch the error behavior, including the impacts of dynamic variations and INS sensor errors of KF and RTS smoother, during training should be confirmed. The performance of proposed schemes still needs to be verified using other independent data sets, which will be presented in the next section. As indicated in Table 2, the improvement indices are presented by comparing to the “raw” attitude errors of KF and RTS smoother.

## 8. Performance verification of the proposed schemes

**Figure 15.**
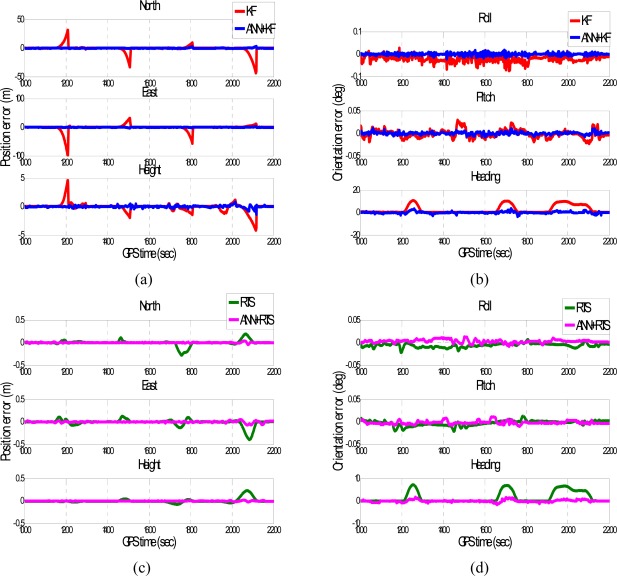
The samples of testing results (Trajectory 1).

**Figure 16.**
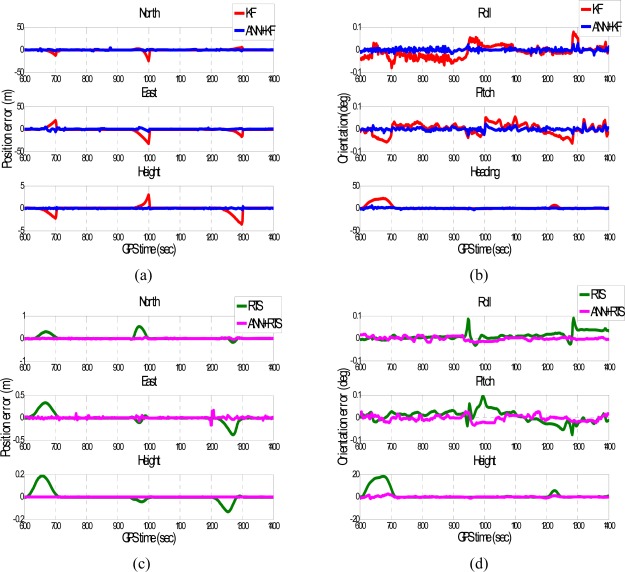
The samples of training results (Trajectory 2).

To verify the performance of the well trained ANN-KF and ANN-RTS smoother schemes, sixteen simulated GPS outages (60 seconds each), which were independent from of the ten outages used for training, were selected and marked in blue circles in each test trajectory based on their dynamic variations, as shown in Figures 11 and 12. Since each field test was conducted independently, the simulated GPS outages selected from the first and second field test used for the verification were independent of those selected from the third field test used for training. The dynamic variations experienced by the vehicle during the simulated outages include straight line segments, sharp turns, accelerations and decelerations. Figures 15 and 16 depict the testing results of the proposed schemes. As shown in the figures, both ANN-KF and ANN-RTS smoother schemes are capable of reducing the POS errors of KF and RTS smoother, respectively.

A summary of the testing results is shown in Table 3. The proposed ANN-KF scheme improves all the errors of roll angles, pitch angles, and heading angles estimated by KF by 80%, 60%, and 80% on average, respectively. In addition, it effectively reduces the positional errors in north, east, and height by 80%, 75% and 80%, respectively. General speaking, the proposed ANN-KF scheme is able to provide significant compensation for the heading errors estimated by KF. This step is very important in mobile mapping applications as heading errors can be considered critical for the positioning errors estimated by KF during GPS outages with the use of low cost MEMS INS/GPS integrated systems.

The proposed ANN-KF compensation scheme is able to improve the accuracies of positional components as well as orientation components in real time. After applying ANN-KF compensation, the orientations estimated by KF can be improved to the level of using RTS smoother in real time mode even with the use of a low cost MEMS IMU. In addition, the proposed ANN-KF scheme can accelerate the in-motion alignment process as well as improve its accuracy.

Table 3 shows the improvements produced by the proposed ANN-RTS smoother scheme. Comparing to the conventional EKF based scheme, the proposed ANN-RTS smoother scheme improves all the errors of roll angles, pitch angles, and heading angles estimated by EKF by 75%, 70%, and 75% on average, respectively. Similarly, the proposed ANN-RTS smoother scheme improves all the errors of orientation components estimated by ANN-KF by 75% on average comparing to the previously developed ANN-KF scheme. In addition, all of the improvements in positional POS parameters reach 80% on average. On the other hand, the proposed ANN-RTS smoother scheme improves all the errors of roll angles, pitch angles, and heading angles estimated by RTS smoother by 65%, 65% and 75% on average, respectively. In addition, all of the improvements in positional POS parameters reach 60% on average.

**Figure 17.**
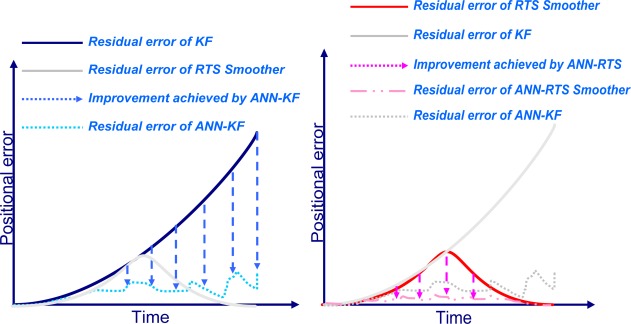
The contribution of proposed ANN-RTS smoother scheme.

Therefore, the proposed ANN-RTS smoother scheme improves all the accuracies of POS parameters estimated by KF and RTS smoother significantly for low cost MEMS based integrated systems. Among the POS parameters compensated by proposed ANN-RTS smoother scheme, the improvement of the orientation parameters is more significant than positional parameters. Figure 17 illustrates the contributions of proposed scheme compared to KF, RTS smoother, and the ANN-KF schemes in terms of the positional error accumulation during GPS signal outages based on the results presented in this study.

Consequently, this study improves the accuracy of POS parameters through evolving the POS algorithms instead of taking the direct route by using a tactical grade IMU or higher. Of course the replacement of a low cost MEMS IMU with a tactical grade IMU or higher can enhance the performance of POS directly, however, the availability of tactical grade IMUs or higher is limited in terms of cost or government regulation.

For low cost MEMS based integrated systems with the proposed ANN-RTS smoother scheme, the accuracies of the POS parameters estimated can be improved to the level of using a medium tactical grade system. Therefore, future inclination of low cost MEMS based integrated systems for land based MMS applications can be anticipated with sufficient accuracies of POS parameters required for direct geo-referencing procedure and with reduced costs for the hardware used.

## 9. Conclusions

This study developed an ANN embedded POS algorithm to reach higher estimation accuracy of POS parameters using a novel procedure that combines an ANN architecture and RTS smoother for post-mission processing. The ANN architectures were first trained to learn the error behavior of the KF and RTS smoother using one of the field data sets collected with a tactical grade INS/GPS integrated system. Then, the well-trained schemes were verified using the rest of the test data sets.

The preliminarily results presented in this article indicate that the proposed ANN-KF compensation scheme is able to improve the accuracies of positional components as well as orientation components in real time. After applying ANN-KF compensation, the orientations estimated by the KF can be improved to the level of using RTS smoother in real time mode even with the use of a low cost MEMS IMU. The proposed ANN-RTS smoother scheme significantly improves all the errors of POS parameters estimated by KF and RTS smoother for MEMS systems. Among the POS parameters compensated by the proposed ANN-RTS smoother scheme, the improvement of the orientation parameters is more significant than that of positional parameters. Consequently, for the low cost MEMS system with the proposed ANN-RTS smoother compensation, the POS parameters estimated by RTS smoother can be improved to the level of using a medium tactical grade system.

This study improves the accuracy of POS parameters through evolving the POS algorithms instead of taking the direct route by using a tactical grade IMU or higher. Of course the replacement of a low cost MEMS IMU with a tactical grade IMU or higher can enhance the performance of POS directly, however, the availability of tactical grade IMUs or higher is limited in terms of cost or government regulation. For low cost MEMS based integrated systems with the proposed ANN-RTS smoother scheme, the accuracies of the POS parameters estimated can be improved to the level of using a medium tactical grade system. Therefore, future inclination of low cost MEMS based integrated systems for land based MMS applications can be anticipated with sufficient accuracies of POS parameters required for direct geo-referencing procedure and with reduced costs for the hardware used.

## Figures and Tables

**Table 1.** The summary of experimental condition.

**Index**	**NVS**			**PDOP**			**speed (m/s)**			**Date**	**Duration (seconds)**
	**Min.**	**Max.**	**Avg.**	**Min.**	**Max.**	**Avg.**	**Min.**	**Max.**	**Avg.**		
**Tj-1**	4	10	7	1.2	5.8	2.2	0	22	7.5	03.17.2005	2,400
**Tj-2**	4	10	7	1.1	5.8	2.1	0	22	8.2	03.17.2005	1,850
**Tj-3**	4	10	7.5	1.4	5.8	2.4	0	22	7.8	03.16.2005	1,700

NVS: Number of visible satellite, Min.: Minimum, Max.: Maximum, Avg.: Average, Speed: Horizontal velocity

**Table 2.** Training results summary.

**Method**	**POS**	**RMS value**		**Improvement. (%)**	
		**Original(KF)**	**Compensated**	**Against KF**	**Against RTS**

Tj-3 (KF + ANN)	North(m)	30.5	1.76	94	--
	East(m)	16.48	1.28	92	--
	Height(m)	3.84	0.31	92	--
	Roll(deg)	0.182	0.025	87	--
	Pitch(deg)	0.384	0.046	88	--
	Heading(deg)	18.433	1.783	90	--

**Method**	**POS**	**Original(RTS)**	**Compensated**	**Against KF**	**Against RTS**

Tj-3 (RTS + ANN)	North(m)	0.44	0.15	99	66
	East(m)	0.37	0.12	99	68
	Height(m)	0.21	0.03	99	86
	Roll(deg)	0.022	0.008	96	64
	Pitch(deg)	0.034	0.012	97	65
	Heading(deg)	1.541	0.3023	98	80

Remarks	--Left blank intentionally				

**Table 3.** Testing results summary.

**Method**	**POS**	**RMS value**		**Improvement (%)**	

		**Original(KF)**	**Compensated**	**Against KF**	**Against RTS**

Tj-1 (KF + ANN)	North(m)	25.28	4.18	79	--
	East(m)	23.15	6.57	72	--
	Height(m)	6.45	0.85	87	--
	Roll(deg)	0.982	0.244	75	--
	Pitch(deg)	0.753	0.283	63	--
	Heading(deg)	48.702	8.043	84	--

Tj-2 (KF+ ANN)	North(m)	28.54	6.21	78	--
	East(m)	23.12	7.58	67	--
	Height(m)	5.12	0.75	85	--
	Roll(deg)	0.782	0.212	73	--
	Pitch(deg)	0.854	0.381	55	--
	Heading(deg)	13.25	2.18	84	--

**Method**	POS	**Original(RTS)**	**Compensated**	**Against KF**	**Against RTS**

Tj-1 (RTS+ ANN)	North(m)	0.43	0.18	99	58
	East(m)	0.32	0.15	99	53
	Height(m)	0.23	0.08	99	65
	Roll(deg)	0.543	0.125	87	78
	Pitch(deg)	0.325	0.083	89	74
	Heading(deg)	24.325	3.854	92	84

Tj-2 (RTS + ANN)	North(m)	0.38	0.17	99	55
	East(m)	0.34	0.13	99	62
	Height(m)	0.27	0.09	99	67
	Roll(deg)	0.256	0.103	81	60
	Pitch(deg)	0.312	0.124	85	61
	Heading(deg)	4.875	1.235	89	75

Remark	--Left blank intentionally				
